# Gastrointestinal Bleeding Unmasking Gastric Metastases From a Primary Breast Malignancy: A Case Report

**DOI:** 10.1155/crgm/2763114

**Published:** 2025-05-03

**Authors:** Thilini Delungahawatta, Richard Hum, Stephanie M. Woo, Rashmi Samdani, Mark J. Real

**Affiliations:** ^1^Department of Internal Medicine, MedStar Union Memorial Hospital, Baltimore, Maryland, USA; ^2^Georgetown University School of Medicine, Washington, D.C., USA; ^3^Department of Gastroenterology, MedStar Georgetown University Hospital, Washington, D.C., USA; ^4^Department of Pathology, MedStar Georgetown University Hospital, Washington, D.C., USA

**Keywords:** anticoagulation, gastrointestinal bleed, gastrointestinal metastatic disease, metastatic breast cancer, mixed breast cancer, stomach neoplasm

## Abstract

Metastases to the gastrointestinal tract from primary breast malignancies are rare. Acute gastrointestinal bleeding in patients with history of breast cancer, however, should raise clinical suspicion and warrant further investigation for metastatic disease involving the gastrointestinal tract. We report a case of a 74-year-old female with metastatic breast cancer and provoked thromboembolic events on anticoagulation, who was found to have poorly cohesive gastric carcinoma with immunohistochemistry consistent with primary breast malignancy, after presenting with new-onset melena. Use of anticoagulation may have exacerbated bleeding prompting endoscopic examination. Biopsy and histologic assessment are needed for definitive diagnosis and timely management.

## 1. Introduction

Breast cancer is the most common malignancy in women globally, with metastases most often involving the bone, lung, liver, and brain [1]. Metastases to the gastrointestinal (GI) tract however are rare, with reported incidence of less than 1%, among which 28% include gastric lesions [2]. Symptoms arising from gastric metastases are nonspecific and often attributed to adverse effects of concomitant chemotherapy or radiotherapy [3]. Endoscopic appearance is also variable, with lesion descriptions ranging from microscopic infiltration to nodules, polyps, gross ulceration, or hypertrophy, thereby necessitating histological examination for definitive diagnosis [4]. Given the rarity and clinical ambiguity of gastric metastases originating from breast cancer, diagnosis is often delayed, and prognosis remains poor [4]. Herein, we report a case involving a patient with mixed lobular and ductal, estrogen receptor (ER) positive, progesterone receptor (PR) negative, and human epidermal growth factor receptor (HER2) negative, metastatic breast cancer presenting with melena, unmasking further metastatic disease to the stomach.

## 2. Case Presentation

A 74-year-old African American female with metastatic breast cancer to bone and liver (ER-positive, PR-negative, and HER2-negative) no longer a candidate for palliative cytotoxic chemotherapy given compromised performance status and history of multiple pulmonary and venous thromboembolism events on apixaban presented to the Emergency Department with a three-day history of melena. She described multiple episodes of dark, tarry stools with nausea and progressively worsening epigastric abdominal pain that was sharp in nature and radiated across her upper abdomen. She also noted one episode of epistaxis which spontaneously resolved. She had not experienced prior bleeding per rectum, but she did have symptomatic anemia requiring intermittent blood transfusions while on chemotherapy. She denied any hematemesis, hematuria, chest pain, or light-headedness. She had been on anticoagulation since 2013 for thromboembolism prophylaxis but had switched from warfarin to apixaban about one year prior due to supratherapeutic INR. The apixaban was held since onset of melena, and the patient was not on any other known anticoagulation or steroids, or NSAIDs.

With regard to the breast cancer, she was diagnosed with stage IIIC, grade 2, ER-positive, PR-negative, and HER2-negative adenocarcinoma of the right breast with ductal and lobular morphology in 2014 and underwent right radical mastectomy. Shortly after diagnosis, she was found to have metastasis to the bone (sternum, thoracic spine, lumbar spine, and pelvis) and was started on chemotherapy. In 2020, she was found to have metastasis to the liver, and in 2022, there was notable disease progression and metastasis to the bone marrow. She started palliative chemotherapy with paclitaxel, complicated by neutropenia and thrombocytopenia. Her last session was two months prior to presentation.

On presentation, the patient was vitally stable (temperature 36.5°C, heart rate of 66 beats per minute, respiratory rate of 18 breaths per minute, blood pressure 127/69 mmHg, and oxygen saturation of 98% on room air). Laboratory values were significant for anemia (hemoglobin, 7.2 g/dL (reference range 12.5–16.5 g/dL)), low hematocrit (21.2% (reference range 37.5%–49.5%)), leukopenia (3.1 k/μL (reference range 4.0–10.8 k/μL)), and thrombocytopenia (104 × 10^9^/L (reference range 145–400 × 10^9^/L)). Coagulation panel revealed prolonged INR (1.9 (reference range 0.8–1.2)) as well as prothrombin time (17.1 s (reference range 11.8–14.6 s)). Furthermore, liver function tests revealed an elevated total bilirubin (5.1 mg/dL (reference range 0.2–0.9 mg/dL)), aspartate aminotransferase (199 U/L (reference range 0–33 U/L)), and alanine aminotransferase (67 U/L (reference range 10–49 U/L)). Lipase level was also elevated (695 U/L (reference range 12–53 U/L)). Physical exam was largely unremarkable except for mildly distended abdomen, which was nontender to palpation.

**Figure 1:**
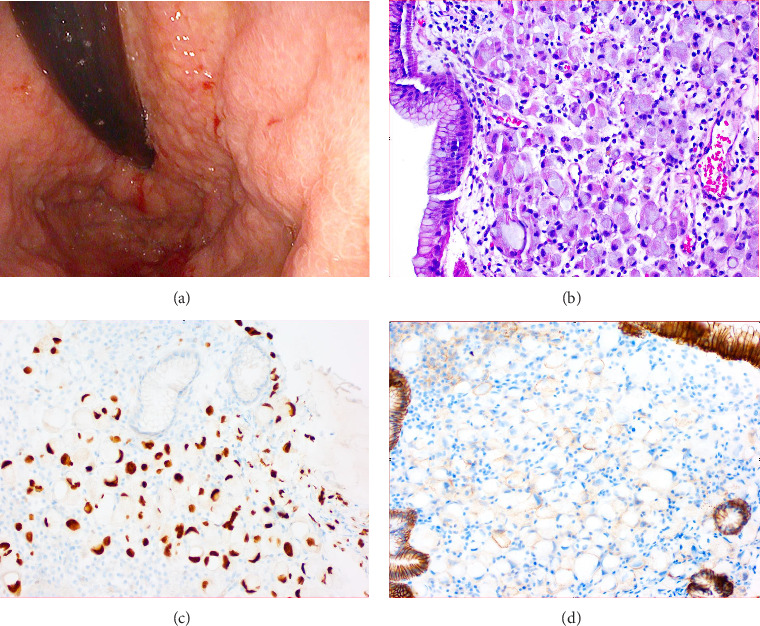
Upper endoscopy showing (a) retroflexed visualization of nonbleeding friable and nodular mucosa in the gastroesophageal junction and gastric cardia with corresponding gastric mucosa biopsy and histopathology showing (b) poorly cohesive gastric carcinoma encompassing signet ring cell histology (H&E stain, magnification ×20) with (c) loss of E-cadherin expression (magnification ×20) and (d) GATA3 positivity (magnification ×20).

The patient received three units of packed red blood cell transfusion with appropriate response. A computed tomography scan of the abdomen was performed which demonstrated cirrhotic liver with hepatic metastasis as well as ascites. An esophagogastroduodenoscopy was performed the following day which showed nonbleeding friable nodular mucosa in the gastric cardia, body, and incisura with loss of normal folds (Figure 1(a)). She had no varices. Biopsy and histopathology examination confirmed poorly cohesive gastric carcinoma, encompassing signet ring cell histology, with positive immunostaining for CK7 and GATA-3 (negative for CK20 and CDX2), consistent with a primary breast source (Figure 1(b)).

The patient decided not to pursue further treatment and opted for palliative care. Hemoglobin remained stable after another unit of blood. Apixaban was resumed at discharge.

## 3. Discussion

Gastric metastases from primary breast cancer are a rare occurrence [5]. In one large nonautopsy case series of women with metastatic breast cancer, 0.3% of patients were found to have metastatic disease to the GI tract, with gastric metastases specified in 28% of these cases [2]. In another study by Taal et al. [6], from a pool of 16,000 endoscopies in patients with breast carcinoma, 51 cases (0.3%) demonstrated gastric metastases, often with features of linitis plastica. These authors also report greater incidence of lobular breast carcinoma compared with ductal carcinoma spreading to the GI tract [2, 6]. The possible pathways for such metastases can involve hematogenous, lymphatic, and/or direct tumor invasion [4]. The estimated interval time between primary breast cancer diagnosis and gastric metastases is four to ten years [7]; however, diagnosis may be delayed given the absence of pathognomonic signs and symptoms. Abdominal pain is the most reported symptom, followed by nausea, vomiting, and weight loss [7]. Data from large series of breast cancer patients have previously suggested melena to be an uncommon presenting symptom of metastatic breast cancer [8]. However, in a recent study involving a Japanese population, three of 11 patients identified to have metastatic gastric tumor originating from breast cancer presented with melena (27.3%) [9]. In our patient, the melena may have been exacerbated by use of apixaban in addition to thrombocytopenia secondary to chemotherapy.

When endoscopic evaluation is pursued, findings can vary between noticeable nodular projections or ulceration to microscopic infiltration of deeper layers [4]. In the latter, diagnosis is often missed with conventional endoscopy and biopsy techniques and requires deep tissue biopsy for identification of tumor cells. The first-line therapy for gastric metastasis from primary breast cancer is chemotherapy with or without hormonal blockade. However, in cases where the primary tumor is well controlled and the gastric tumor is singular, surgery may also be an option [7]. Nevertheless, the mean survival reported in literature for patients with GI metastasis was around seven months [2].

Though rare, our case highlights the need to consider gastric metastases in patients with breast cancer, especially when presenting with new GI symptoms, such as melena or hematochezia. Gastric metastases can have variable clinical presentations and appearance on radiography or endoscopy. Early histologic investigation via biopsy is needed for definitive diagnosis and therapeutic efficacy.

## Data Availability

All data used to support the findings of this case report are available as part of the article and cited references.
